# Central Retinal Artery Occlusion With Triple Cilioretinal Artery Sparing

**DOI:** 10.7759/cureus.48157

**Published:** 2023-11-02

**Authors:** Vaibhav Bhatt, Shrutanjoy Das, Shweta Parakh, Abhijaat Chaturvedi, Amarpal S Gulati, Gaurav Luthra, Saurabh Luthra

**Affiliations:** 1 Ophthalmology, Drishti Eye Institute, Dehradun, IND; 2 Cardiology, Synergy Institute of Medical Sciences, Dehradun, IND

**Keywords:** oct angiography, percutaneous transluminal coronary angioplasty, cilioretinal artery sparing, triple cilioretinal artery, central retinal artery occlusion

## Abstract

We report a rare case of central retinal artery occlusion (CRAO) with triple cilioretinal artery sparing in a 76-year-old male with hypertension who presented with sudden diminution of vision in the left eye (OS) for one day. Optical coherence tomography angiography (OCTA) demonstrated the presence of three cilioretinal arteries and the absence of flow signals in the rest of the macula. Primary ophthalmic treatment was instituted immediately in the form of ocular massage, and acetazolamide 500 mg per oral (PO) stat was given. Systemic investigations revealed a significant blockage in coronary circulation on coronary angiography and an atheromatous plaque at the origin of the left internal carotid artery with 50% stenosis on digital subtraction angiography. Systemic anticoagulants and lipid-lowering agents (statins) were initiated by the cardiologist. Percutaneous transluminal coronary angioplasty was subsequently performed.

At the eight-week follow-up visit, best-corrected visual acuity had improved to 2/60 OS. Fundus examination of the OS revealed optic disc pallor with normal retinal background. Spectral-domain optical coherence tomography showed diffuse retinal thinning except in the area supplied by the three patent cilioretinal arteries. En face OCTA OS showed restoration of retinal flow signal in the macula.

Non-invasive imaging (OCTA) is critical in establishing early diagnosis and initiating prompt treatment in this ocular emergency with underlying potentially life-threatening systemic associations.

## Introduction

Central retinal artery occlusion (CRAO) is caused by obstruction of the central retinal artery, most commonly due to emboli originating from atheromatous plaques in the cardiac valves and carotid arteries, resulting in retinal ischemia [1]. The common presentation of CRAO is painless, monocular acute vision loss with counting fingers vision or worse. The cilioretinal artery, supplied by the posterior ciliary arteries, is a terminal artery perfusing the papillomacular bundle. Although the presence of a cilioretinal artery has been reported in as high as 49.5% of patients, arteries with macular collateral circulation are found in only approximately 15% of the population [2,3]. These are associated with better long-term visual outcomes.

Herein, we report a unique case of CRAO with sparing of three cilioretinal arteries, possibly the first of its kind.

## Case presentation

**Figure 1:**
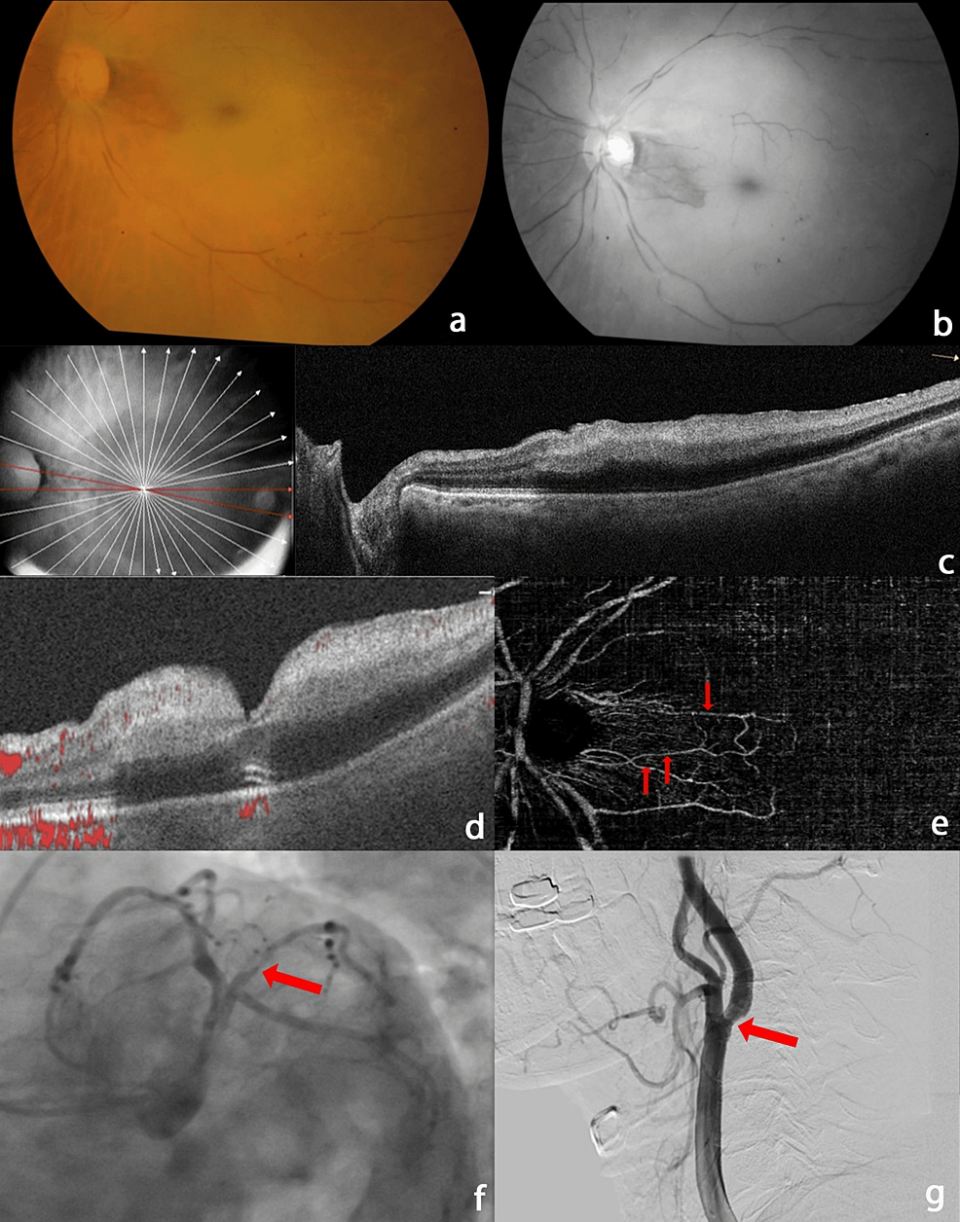
Multimodal imaging of left eye central retinal artery occlusion with triple cilioretinal artery sparing at initial presentation. (A, B) Color and red-free fundus photo showing arteriolar narrowing with segmental blood flow (box-carring), diffuse background pallor with a small area of perfused retina temporal to the optic disc. (C) Spectral-domain optical coherence tomography showing diffuse retinal thickening and inner retinal hyperreflectivity except in the area corresponding to the patent cilioretinal arteries. (D) Optical coherence tomography angiography image showing the absence of flow signals except in the area supplied by patent cilioretinal arteries. (E) En face optical coherence tomography angiography of the disc and macula showed the presence of three cilioretinal arteries (red arrows) and flow void in the remaining vasculature. (F) Left coronary angiogram in spider view showing normal left main coronary artery trifurcating into large type III left anterior descending, nondominant left circumflex, and large ramus intermedius artery with significant (>70%) lesion proximally (red arrow). (G) Left lateral view digital subtraction angiography of carotid arteries showing atheromatous plaque at origin of left internal carotid artery with 50% stenosis (red arrow).

A 76-year-old male with a history of hypertension (controlled on anti-hypertensive medication) presented with painless, sudden diminution of vision in the left eye (OS) for one day. The best-corrected visual acuity (BCVA) was 6/9 in the right eye (OD) and counting fingers close to face (CFCF) in the OS. A relative afferent pupillary defect in the OS was noted. Intraocular pressure was normal in both eyes (OU) (OD 14 mmHg, OS 16 mmHg). Anterior segment examination was unremarkable OU. Fundus examination OD was normal. OS showed a normal-appearing optic disc with a cup-to-disc ratio of 0.3:1, arteriolar narrowing with segmental blood flow (box-carring), and diffuse background pallor with a small area of normal-appearing retina temporal to the optic disc, corresponding to patent cilioretinal arteries (Figures 1A, 1B). Spectral-domain optical coherence tomography (SD-OCT) (RTVue XR Avanti, Optovue, Inc., Fremont, CA) showed diffuse retinal thickening and inner retinal hyperreflectivity except in the small area corresponding to the patent cilioretinal arteries (Figure 1C). Optical coherence tomography angiography (OCTA) (AngioVue, RTVue XR Avanti, Optovue, Inc., Fremont, CA) showed an absence of flow signals in superficial, deep, and outer retinal plexuses except in a well-demarcated area nasal to the fovea (Figure 1D). OCTA of the disc and macula OS showed the presence of three cilioretinal arteries and flow void in the rest of the vasculature (Figure 1E).

A clinical diagnosis of CRAO with triple cilioretinal artery-sparing OS was made. Primary treatment was immediately instituted in the form of ocular massage, and acetazolamide 500 mg per oral (PO) stat was given. Systemic investigations revealed mildly deranged glycated hemoglobin (HbA1c) at 6.7% with a deranged lipid profile (total cholesterol = 268 mg/dl, serum triglycerides = 324 mg/dL) and renal function test (serum creatinine = 1.4 mg/dL). The patient was referred to the cardiology and neurology specialists to rule out systemic co-morbidities and any potential sources of thrombi or emboli. He underwent coronary angiography, which revealed significant (>70%) blockage in the left proximal ramus intermedius artery (Figure 1F). Digital subtraction angiography of carotid arteries showed atheromatous plaque at the origin of the left Internal carotid artery with 50% stenosis (Figure 1G). Percutaneous transluminal coronary angioplasty (PTCA) to large OM1 (obtuse marginal artery) was subsequently performed. Anti-hypertensive medications were continued in the form of metoprolol 12.5 mg PO, amlodipine 5 mg PO, and chlorthalidone 6.25 mg PO. Statins (rosuvastatin 20 mg PO), clopidogrel 75 mg PO, and aspirin 75 mg PO were started by the cardiologist.

**Figure 2:**
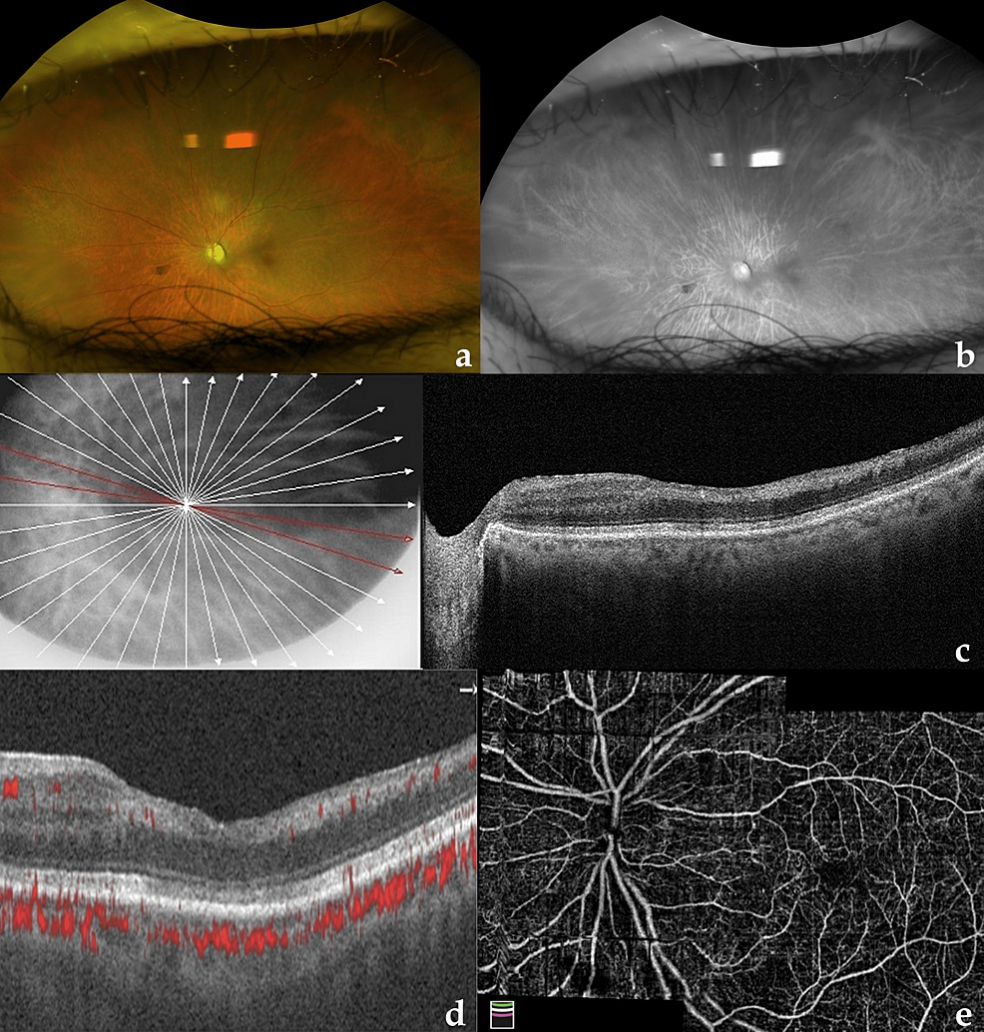
Multimodal imaging of the left eye at eight-week follow-up visit. (A, B) Ultra widefield color and red-free images showing optic disc pallor with arteriolar attenuation. (C) Spectral-domain optical coherence tomography showing diffuse retinal thinning except in temporal juxtapapillary retina. (D) Optical coherence tomography angiography (OCTA) image showing restoration of flow signals in the previously ischemic macula. (E) En face OCTA montage of disc and macula showing restoration of flow in the retinal vasculature.

The vision had improved to 2/60 OS after eight weeks. Fundus examination of the OS revealed optic disc pallor with arteriolar attenuation with a normal retinal background (Figures 2A, 2B). SD-OCT showed diffuse retinal thinning at the posterior pole except for the area supplied by cilioretinal arteries (Figure 2C). OCTA of the macula showed restoration of flow signal in superficial, deep, and outer retinal plexuses (Figure 2D). En face OCTA montage of the disc and macula showed reperfusion of flow void areas noted at the first visit (Figure 2E).

At the latest follow-up visit at six months, the patient maintained status quo OU. Anti-hypertensive drugs, statins, and systemic anticoagulants continued to be administered under the supervision of the cardiologist.

## Discussion

CRAO is not only an ophthalmic emergency but also a medical emergency due to its potentially life-threatening systemic associations such as carotid artery disease and coronary artery disease [2]. The incidence of CRAO has been cited as 0.85 to 1/100,000 persons per year [4]. Four types of CRAO have been described: non-arteritic, non-arteritic with cilioretinal artery sparing, arteritic, and transient non-arteritic [5]. Early clinical findings of CRAO include cherry-red spot (90%), retinal opacity in the posterior pole (58%), retinal arterial attenuation (32%), optic disc edema (22%), and pallor (39%) [2].

Cilioretinal arteries are defined as retinal artery branches, not continuous with the central retinal artery [6]. They make an almost 180-degree hook as they emerge from underneath the retinal pigment epithelium at the rim of the optic disc [6]. Cilioretinal arteries have been shown to originate from the short posterior ciliary arteries and, in rare cases, directly from the choroidal vessels [7]. In a study of 2000 eyes by Justice et al. using stereo-color fundus photography and fluorescein angiography, 32.1% of eyes had one or more cilioretinal arteries [8]. Schneider et al. [9] in their major systematic review of 19 studies (involving 15611 patients) and a prospective cross-sectional observational study of 1000 healthy Caucasians found the per eye prevalence of cilioretinal arteries to be 22.75%. In their study population, the maximum reported number of cilioretinal arteries in one eye was three. The reported prevalence of one cilioretinal artery was 86.81%, two cilioretinal arteries was 11.87%, and three cilioretinal arteries was 1.32% [9].

Macular supply is defined as the cilioretinal artery reaching a circle centered on the fovea with a radius of fovea-to-optic disc rim distance minus one-disc diameter [9]. Macular supply was observed in a high percentage (76.05%) of patients with temporal cilioretinal arteries, i.e., 381 out of 501 patients [9]. The presence of a patent temporal cilioretinal artery that provides macular supply in CRAO is usually a good prognostic factor for visual outcome as shown in a study of 260 eyes with CRAO by Hayreh et al. [10]. They found that 35 (13.46%) eyes had cilioretinal sparing. Also, a higher rate of improvement of visual acuity within seven days was seen in cases of CRAO with cilioretinal artery sparing (67%) compared to cases of CRAO without any cilioretinal artery (22%) [10]. Timing of reperfusion is critical for visual recovery with reports of no permanent retinal damage and return to normal vision if the retinal circulation is restored within an hour of insult [11]. In our case, at the eight-week follow-up, though there was an anatomical recovery of perfusion in the macula, visual acuity remained poor due to a lack of macular supply by the cilioretinal arteries. After an extensive literature search, we have come across a singular case report of a double cilioretinal artery sparing CRAO [12].

## Conclusions

To the best of our knowledge, ours is the first case report of CRAO with triple cilioretinal artery sparing. Invasive and relatively time-consuming investigations such as fluorescein angiography were not performed at presentation due to the emergent nature of the clinical presentation and the presence of potential unknown systemic risk factors such as nephropathy. OCTA provided a crucial advantage in this emergency owing to its fast acquisition time and non-invasive nature. Prompt systemic evaluation led to the timely diagnosis and treatment of the underlying ominous coronary and carotid artery disease.
